# Morphology of Shear-Induced Polymer Cylindrites Revealed by 3D Optical Imaging

**DOI:** 10.1021/acs.macromol.2c01433

**Published:** 2022-11-10

**Authors:** Shu-Gui Yang, Liang-Qing Zhang, Jiaming Cui, Xiang-bing Zeng, Baolin Guo, Feng Liu, Goran Ungar

**Affiliations:** † Shaanxi International Research Center for Soft Materials, State Key Laboratory for Mechanical Behaviour of Materials, Xi’an Jiaotong University, Xi’an710049, China; ‡ College of Material Science and Engineering, Xi’an University of Science and Technology, Xi’an710054, China; § Department of Materials Science and Engineering, University of Sheffield, SheffieldS1 3JD, U.K.; ∥ State Key Laboratory for Mechanical Behavior of Materials, Frontier Institute of Science and Technology, Xi’an Jiaotong University, Xi’an710049, China

## Abstract

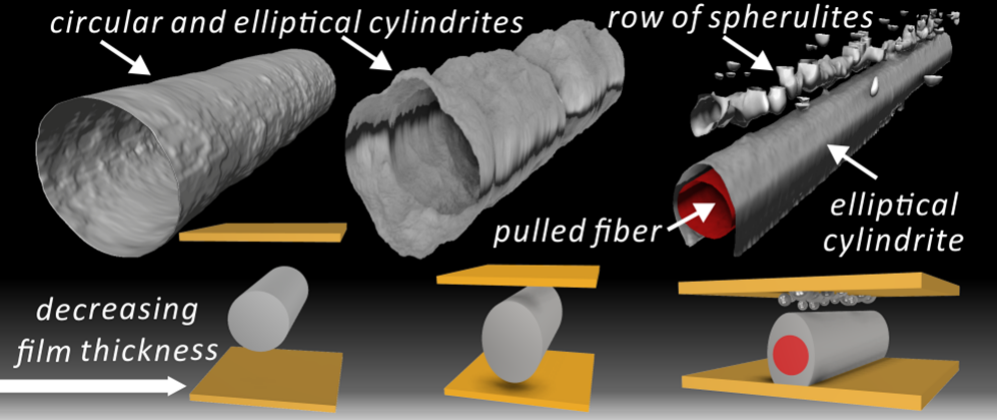

Two-photon confocal laser microscopy was used to obtain three-dimensional (3D) images of the morphology of poly(lactic acid) after shear-induced crystallization. The necessary fluorescence contrast was achieved by doping the polymer with Nile Red. The dye gets partially rejected from the growing crystalline aggregates during their formation, thus creating a renderable high-low fluorescence boundary outlining the shape of the aggregates. Parallel-plate melt-shearing and pulling a glass fiber through the melt were used as the two methods to achieve shear-induced crystallization. This study focuses on the shape of the resulting cylindrites, i.e., large-diameter shish-kebabs. The first 3D images of polymer cylindrites show that, if far from boundaries, they are circular cylinders, highly regular after fiber pull, but less so after parallel-plate shear. In the latter case, the cylindrite reveals the trajectory of the foreign particle that had nucleated its growth. Interestingly, lateral growth of the cylindrites was found to accelerate toward the sample surface when approaching it, giving the cylindrite an elliptical cross section. Furthermore and surprisingly, in the case of fiber pull, a row of spherulites is nucleated at the polymer–substrate interface nearest to the fiber, aligned along the fiber axis and appearing ahead of the rest of the space-filling spherulites. Both the phenomena, elliptical cylindrites and row of spherulites, are attributed to negative pressure buildup peaking at the cylindrite growth front and at the nearby film surface caused by crystallization-induced volume contraction. The pressure and flow distribution in the system is confirmed by numerical simulation. The results illustrate the value of 3D imaging of crystalline morphology in polymer science and polymer processing industry.

## 1 Introduction

As the largest group of commercial polymeric materials, semicrystalline polymers (SCPs) are known to form aggregates of their thin lamellar crystals, such as spherulites,^1−5^ axialites,^6−8^ transcrystalline layers,^9−12^ row-nucleated structures also known as shish-kebabs,^13−18^ and cylindrites.^19−21^ Crystalline morphology directly affects the performance of SCPs, primarily mechanical but also optical, barrier properties, etc.^22−24^

So far, the morphology of SCPs has been studied mainly using polarizing optical microscopy (POM),^25−27^ atomic force microscopy (AFM),^28−30^ and transmission and scanning electron microscopies (TEM, SEM).^31−33^ These methods are limited to thin films or sections of the bulk and only give two-dimensional (2D) images. Generally, the features in the third dimension have been assumed rather than observed. To get 3D information, in rare cases, sectioning and reconstruction were performed with limited reliability. Recently, successive layer ablation combined with SEM was employed as a means to visualize bicontinuous phases in block polymer.^34,35^ Electron tomography has been applied in a few cases,^36^ but this technique is limited to small scale, e.g., a fraction of spherulite, and suffers from problems of radiation damage. In X-ray computer tomography (XCT), like in medical CT and electron tomography, a three-dimensional (3D) image is reconstructed from a set of absorption images recorded at different sample tilts. XCT has been highly successful recently, particularly in polymer fiber composites, where individual fibers and voids could be well resolved.^37,38^ X-ray phase tomography, on the other hand, is based on the weak contrast in X-ray refractive index, producing an X-ray phase shift detectable when coherent synchrotron radiation is used. Polymer blends and foams have been studied.^39−41^ Confocal laser microscopy has also been used in the 3D imaging of polymer blends and block copolymers.^42,43^ All these methods rely on the difference in chemical composition. However, the crystalline and amorphous phases share the same chemical composition, so chemical contrast is not available for imaging the morphology of single-component SCPs. An even bigger challenge is the visualization of the outlines of crystalline aggregates, such as spherulites, in fully crystallized polymers. SCP morphology on the super-μm scale has not been reported until our recent work in which we use two-photon confocal laser scanning microscopy (2PCM) on suitably labeled isotactic polypropylene (iPP) and poly(lactic acid) (PLA).^44,45^ The key step was the addition of a compatible fluorescent dye that segregated at spherulite boundaries either during crystallization or through postcrystallization penetration of the dye solution. In the 2PCM study, spherulites have been seen for the first time in 3D, both in neat polymers and in their composites with inorganic nanoparticles. The study revealed previously unsuspected morphologies such as “vases” and “goblets” as well as nonspherical “spherulites”.^45^ These first results have uncovered unfamiliar modes of self-assembly in familiar SCPs and opened new perspectives on a polymer microstructure.

No less important than spherulites are shish-kebabs and cylindrites that are formed by crystallization from flowing melt and are the basic morphology of melt-spun fibers, blown films, and many thermoplastic composites.^46^ The term “shish-kebabs” was introduced by Keller to describe fibrous structures formed in stirred polymer solutions,^14^ a morphology first reported by Pennings.^46,47^ Shish-kebabs were shown to contain a central core fiber along the flow direction (the “shish”) and chain-folded lamellae (the “kebab”) grown epitaxially normal to the fiber.^46,48,49^ While the term shish-kebab originates from the morphology from stirred solution, the structure had in fact been identified in the bulk considerably earlier when Keller postulated them to explain the unusual “*a*-axis” crystalline orientation from X-ray diffraction alone.^13,50^ The shish is brought about by the row of nuclei induced by flow or stress in the melt. Their high linear density of nuclei means that the initiated lamellae are confined to growth normal to the shish rather than splaying out as they would do from a point nucleus in axialites and spherulites. At high shear rates, this kind of 2D growth usually stops after a couple of μm as the closely spaced shish-kebabs collide. Alternatively, in more quiescent conditions, the 2D growth can continue to form cylindrical objects tens of microns in diameter, which are nowadays known as cylindrites. Considerable effort has been devoted to their morphological studies.^19,20,50−52^ 3D picture of such cylindrites and their surrounding is still missing. Could the third dimension of cylindrites be affected by surface effects as we already saw in spherulites? Are the cylindrites really cylindrical?

In this work, we study cylindrites of suitably labeled PLA by confocal fluorescence microscopy. Growth of the cylindrites is induced by two methods: sliding parallel plates and fiber pull.^53^ Polymer cylindrites are successfully displayed in 3D for the first time. In the bulk, their cylindrical symmetry is indeed verified. However, in the shear experiments, that symmetry is broken close to the polymer–substrate interface. In the case of a fiber pull, it is what happens in the surrounding of the cylindrite that is most intriguing. Among other things, the study highlights the role of negative pressure in polymer crystallization and its role in affecting crystalline morphology.

## 2 Experimental Section

### 2.1 Materials

PLA 4032D containing around 2% d-lactide (*M*_w_ = 2.23 × 10^5^ g/mol and *M*_n_ = 1.06 × 10^5^ g/mol) was provided by NatureWorks (USA). The Nile Red dye (NR, C_20_H_18_N_2_O_2_) and 1,4-dioxane (C_4_H_8_O_2_) were purchased from Sigma-Aldrich (USA). All chemicals were analytical grade and used as received without further purification. The glass fiber with a diameter of ∼10 μm was used without surface modification.

### 2.2 Sample Preparation

The freeze-drying method was employed to prepare a uniform mixture of PLA and NR. First, PLA pellets and NR powder in a weight ratio of 2000:1 were dissolved in 1,4-dioxane with the aid of mild stirring at 50 °C. Then, the solution was dropped in liquid nitrogen for quick freezing. After solidification, a vacuum was applied to sublimate the solid solvent at 0 °C till the solvent was completely removed.

**Figure 1:**
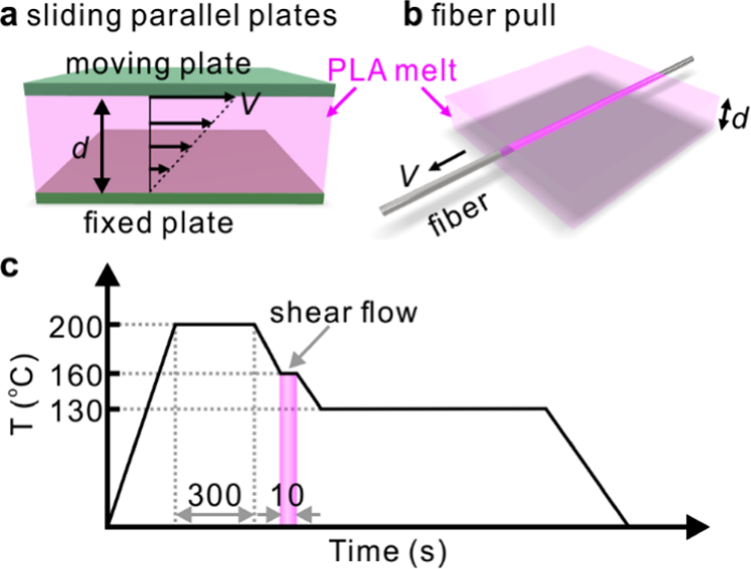
Schematic representation of the flow geometries: (a) sliding parallel plates and (b) fiber pull. (c) Temperature-shear protocol.

Two different flow geometries have been used to induce cylindrites in NR/PLA film: sliding parallel plates (Figure 1a) and fiber pull (Figure 1b). The preparation procedures were as follows (Figure 1c): The NR/PLA blends were heated and annealed at 200 °C in nitrogen for 300 s to erase thermal history. Afterward, the sample was cooled to 160 °C at 30 K/min. Once that temperature was reached, shear was applied by either sliding the cover glass or by pulling the buried glass fiber. The duration of the shear was about 10 s. The sample was then rapidly cooled to 130 °C and kept at that temperature for different times. Finally, it was quenched to room temperature. The sliding speed of cover glass (*v*) is about 1 mm/s, and the corresponding shear rate (γ̇) can be calculated by the following equation
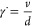
1where *d* is the thickness of PLA melt. For sliding cover glass experiments, the shear rate is 10–50 s^–1^ depending on the thickness of the PLA melt. For fiber pull experiments, the pull speed of the fiber, *v* is also ∼1 mm/s, and the corresponding shear rate at the interface can be calculated using the following equation proposed by Monasse^54^
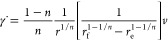
2where *r*_f_ and *r*_e_ are the half-thickness of the PLA melt and fiber radius, respectively. *r* is the distance from the fiber axis and *n* is the exponent of the rheological power law equation, which is 0.3 as obtained from small-amplitude oscillatory shear measurements on the same grade PLA at 160 °C.^55^ For the fiber pull experiments, the shear rates were above 500 s^–1^ at the interface, while they dramatically decreased upon moving away from the fiber (the calculated shear rate profile of the fiber pull experiments is shown in Figure S1 of the Supporting Information).

### 2.3 Microscopy Methods

Polarized optical microscopy (POM) was performed on an Olympus BX51 microscope (Olympus, Japan) equipped with an Olympus DP74 camera. An LTS420E heating cell with a T95-HS controller (Linkam, UK) was used. Fluorescence microscopy (FM) micrographs were recorded in reflection mode using a CoolLED pE-300 white light source, a BP 460–490 excitation filter, a DM 500 dichromatic mirror, and an LP 520 emission filter.

For 3D imaging, an upright Zeiss LSM 510 META confocal microscope (Zeiss, Germany) equipped with a Ti-Sapphire multiphoton laser was applied to take images at different depths of the sample. The excitation wavelength was 1000 nm, and the fluorescence from NR was collected through a BP 565–615 band pass filter (see Figure S2 in the Supporting Information). A theoretical resolution of 2PCM in the lateral (Δ*xy*) and axial (Δ*z*) directions is ∼300 and ∼1000 nm, respectively (the calculation is shown in Section S3 of the Supporting Information). Resolution is also determined experimentally by imaging a sharp edge of a glass fiber immersed in NR-doped amorphous polystyrene. Based on the full width at half-height (FWHH) of the derivative of the brightness profile normal to the sharp edge, Δ*xy* and Δ*z* for the two-photon microscopy are determined to be 0.35 and 0.84 μm, respectively, and those for the one-photon confocal microscopy to be 0.37 and 1.28 μm, respectively (see also ref (45)). In this work, the red color of FM images has been converted to 256 gray levels to improve visual intensity resolution.

### 2.4 Finite Element Simulation

Using Mathematica, crystallization-induced negative pressure and melt flow around a growing cylindrite is simulated by solving the Navier–Stokes equations in 2D boxes, one of size 6 × 1 (−3 ≤ *x* ≤ 3, 0 ≤ *y* ≤ 1) and the other of size 6 × 0.6 (−3 ≤ *x* ≤ 3, 0 ≤ *y* ≤ 0.6). The boxes are discretized to triangles of area ≤0.0002, with a total of 45 724 and 26 752 triangles respectively. The growing cylindrite is represented by a circular boundary at coordinates (0, *z*), with *z* one-half the height of the box (*z* = 0.5 or 0.3), with a radius of 0.15 and a constant outflow rate at the boundary. Two counterbalancing inflowing boundaries are placed close to the two side edges of the box, at coordinates (±2.5, *z*). The flow rates are set to zero at the four straight boundaries of the box. The equations are solved numerically in iterations until the estimated normalized error is smaller than 10^–14^ for the data set.

## 3 Results and Discussion

In Section 3.1, we describe 2D microscopy results on PLA cylindrites induced by sliding parallel plates and by fiber pull. We compare the FM and POM images and discuss the observed distribution of the dye during the growth of the cylindrite. Having learned from the 2D experiments, we succeed in obtaining 3D images of PLA cylindrites induced by sliding parallel plates; this is described in Section 3.2. In Section 3.3, 3D images of cylindrites induced by fiber pull are displayed together with their intriguing spherulite environment. The factors that affect the 3D morphology of cylindrites are discussed and summarized in Section 3.4.

### 3.1 2D Microscopy Observations

**Figure 2:**
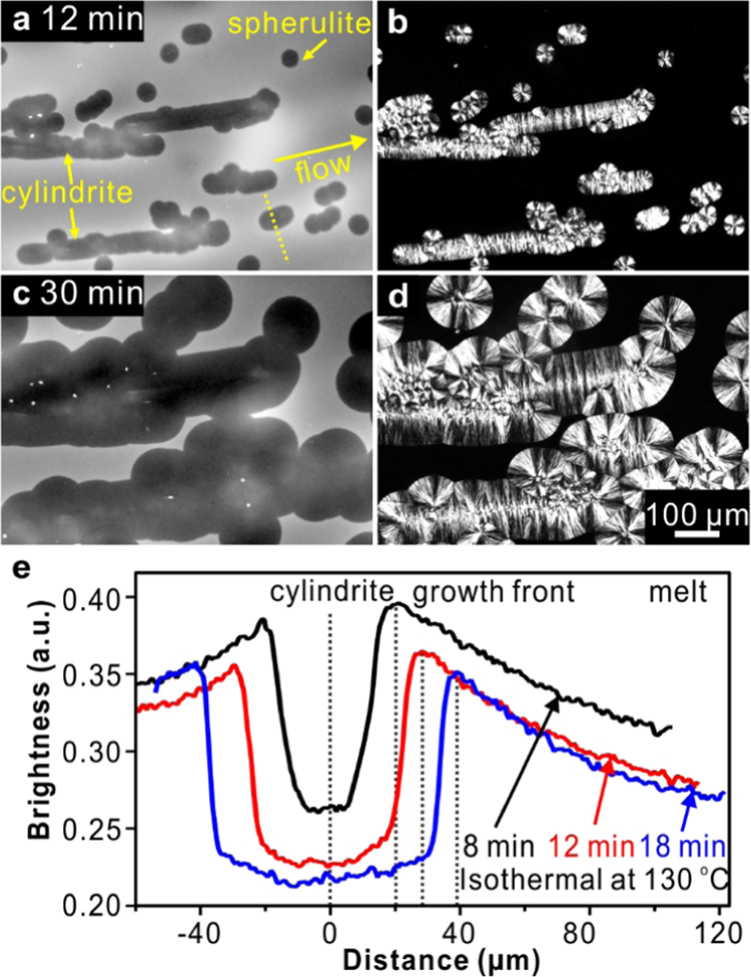
2D micrographs of PLA doped with 0.05 wt % Nile Red isothermally crystallized at 130 °C for (a, b) 12 min and (c, d) 30 min, after shearing between parallel glass plates at 160 °C. (a, c) FM and Nile Red are partially rejected from the growing spherulites and cylindrites, which therefore appear darker in FM. (b, d) POM. (e) Line profiles of fluorescence intensity across the cylindrite and along the yellow dashed line in (a).

Shearing melt by sliding parallel plates is supposed to produce a uniform shear rate everywhere. However, because of the unavoidable presence of foreign inclusions in commercial polymers, a significant increase in shear rate could occur near these foreign particles.^56,57^ Thus, shear flow-induced cylindrites may not form everywhere when a mild shear is applied, and stress-induced crystallization may be induced only locally. Isolated cylindrites can therefore form, while the remaining material crystallizes as spherulites. Similar crystallization behavior has already been reported by Yamazaki et al. in polyethylene and iPP.^58^ Here, the growth of both cylindrites and spherulites of NR-doped PLA are observed after the application of shear (sliding parallel plates), as shown in Figure 2a–d. In FM images (Figure 2a,c), it can be seen that the dark stripes and circles growing within the brighter melt coincide with the birefringent cylindrites and spherulites, respectively (Figure 2b,d). Unlike the spherulites growing radially from the nucleus, the cylindrites grow in width perpendicular to the flow direction due to the large nucleation density along the path of a foreign particle moving under shear flow.

Notice that the brighter rim around the growing cylindrites and spherulites can be seen in FM. The brighter rim is because the dye is pushed ahead of the growth front. Figure 2e displays the line profiles of fluorescence intensity across a PLA cylindrite, where high brightness means a high concentration of the dye. The cylindrite has a lower concentration of the dye than the melt, while at the moving cylindrite boundary, it displays a peak concentration of the dye, which continuously decreases from the boundary into the melt. The distribution of dye during cylindrite growth is determined by the diffusion coefficient of dye in the melt (*D*_m_) and the growth rate of cylindrite (*G*_c_).^59,60^ This phenomenon will be discussed further below.

**Figure 3:**
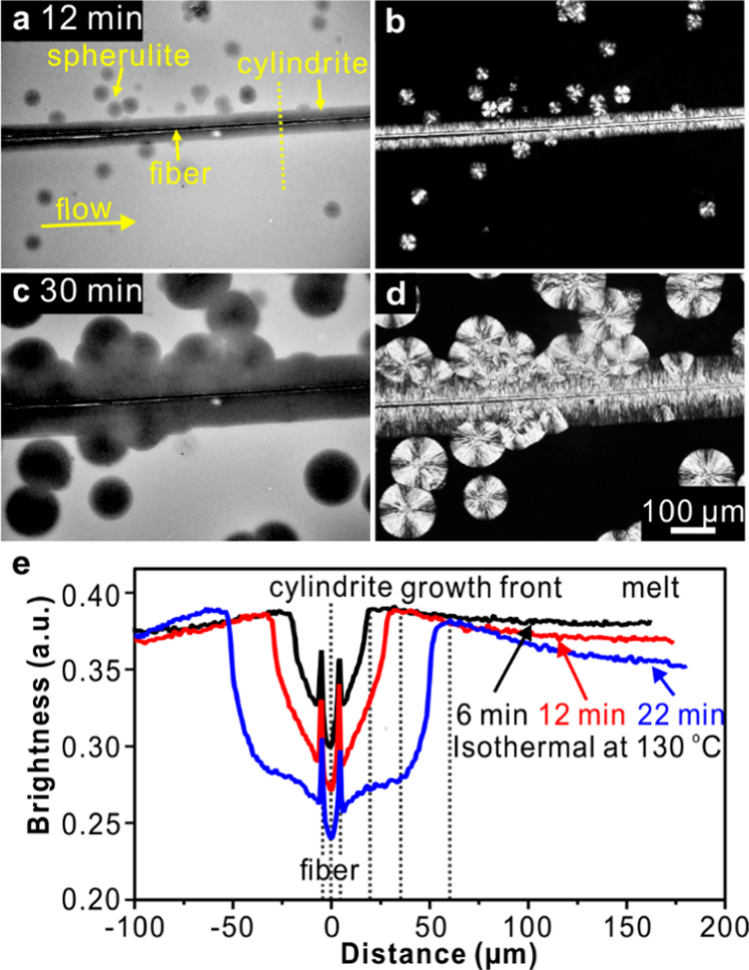
2D micrographs of NR-doped PLA isothermally crystallized at 130 °C for (a, b) 12 min and (c, d) 30 min, after the application of “fiber pull” shear at 160 °C. (a, c) FM and Nile Red are partially rejected from the growing spherulites and cylindrites. (b, d) POM. (e) Line profiles of fluorescence intensity across the cylindrite as indicated by the yellow dashed line in (a).

Pulling a fiber through a polymer melt has the advantage of producing intense and highly localized shear flow. For this reason, fiber pull has been used as a model method for studying flow-induced crystallization.^55,61^ It should be pointed out that the glass fiber used in this work has no nucleating ability for PLA (see Figure S3 in the Supporting Information). In our experiments, after pulling a certain distance, the fiber was left inside the sample when the images were recorded. As shown in Figure 3a–d, FM and POM micrographs show the PLA cylindrite wrapped around the fiber and growing radially. Both the cylindrite and the spherulites are again darker than the surrounding melt in FM images. The fiber is even darker than either the cylindrite or the spherulites, as there is no dye inside it.

Figure 3e shows the line profiles of fluorescence intensity across the cylindrite. As discussed before, the distribution of dye is decided by the dye diffusion coefficient *D*_m_ and the growth rate *G*_c_. Here, *D*_m_ was measured by tracing the time evolution of the concentration profile of a binary PLA melt system with a preset initial concentration step (the results are reported elsewhere). The measured *D*_m_ is 0.83 μm^2^/s at 130 °C, and the characteristic time required for Nile Red to migrate a distance *s* from the growth front is given by τ_D_*= s*^2^/*D*_m_. Meanwhile, *G*_c_ is ∼0.04 μm/s at the same temperature, and the time for a PLA spherulite to grow by *s* is τ_G_*= s*/*G*_c_. Thus, for *s* = 1 μm, τ_D_ and τ_G_ are 1 and 25 s, respectively. Obviously, the dye can move much faster than the growth front of a PLA spherulite at the temperature of the experiment. It would be expected that the excess dye molecules, trapped in the amorphous phase in the spherulite, migrate to the growth front and disperse in the surrounding melt, resulting in a boundary peak of dye concentration.^62^ This agrees with our FM observations.

In addition, there is an overall decrease in fluorescence during the growth of PLA cylindrite, as shown in Figures 2e and 3e. We attribute this to the effect known as aggregation-induced fluorescence quenching, caused by increased intermolecular π–π interaction as the dye molecules, being excluded from the crystalline lamellae, are squeezed into the smaller volume of the amorphous interlamellar layers of the cylindrite.

### 3.2 3D Images of Cylindrites Induced by Parallel-Plate Shear

**Figure 4:**
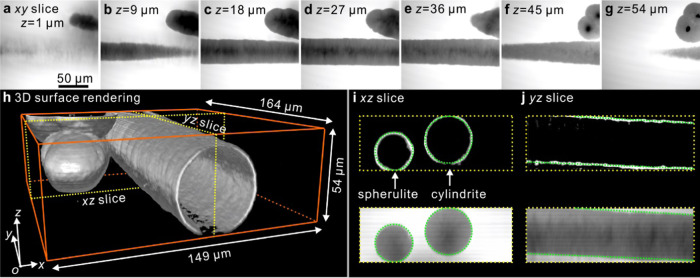
3D images of a PLA cylindrite in a 54 μm film induced by parallel plates shear. After isothermal crystallization at 130 °C for ∼20 min, the sample was quenched in ice water. (a–g) Representative *xy*-slices recorded by two-photon microscopy, bottom to top. (h) 3D surface rendering reconstructed from *xy*-slices. (i) and (j) Vertical *xz*- and *yz*-slices framed by yellow dotted lines in (h), respectively: top row, surface rendering; bottom row, solid rendering.

To obtain 3D images of cylindrites, 2PCM was used to record the *xy*-slices in 1 μm *z*-increments of an NR-doped PLA sheet that had been sheared according to the protocol in Figure 1. Figure 4a–g shows selected *xy*-slices. Similar to the observations with 2D FM, the gray horizontal stripe is the cylindrite, and at the top right of it are several colliding spherulites. For 3D imaging, here we apply the surface rendering method where the surface shown connects the points of the highest gradient in fluorescence intensity using the data set of the full range of *z*-slices. As shown in Figure 4h, the outer surface of the cylindrite thus created appears like a round tube. Although cylindrites and shish-kebabs have been studied by microscopies of different kinds for decades, the third dimension has not been seen. Here, the image of the cylindrite clearly shows the outlines in real 3D space and depicts the trajectory of the moving foreign particle that initiated its growth. The image reveals details such as its axis is inclined to the top glass surface by 3.2 ± 0.3°. In a systematic study of flow-induced crystallization, such information may be useful in distinguishing between laminar and turbulent flow and give more detailed clues on the rheology of a real shear-crystallizing system. The complexity of such a system is thought to be augmented by feedback effects from crystallization itself, such as negative pressure and cavitation.

Figure 4i,j shows the *xz*- and *yz-*slices, respectively, as marked by the yellow dotted lines in Figure 4h. The top and bottom rows of Figure 4i,j correspond to the slices from the 3D surface and volume rendering, respectively. In comparison with the dotted green reference circles in Figure 4i and the two parallel dotted lines in Figure 4j, it can be seen that the cylindrite is very close to circular, while its small misalignment relative to the sample surface is somewhat less expected.

**Figure 5:**
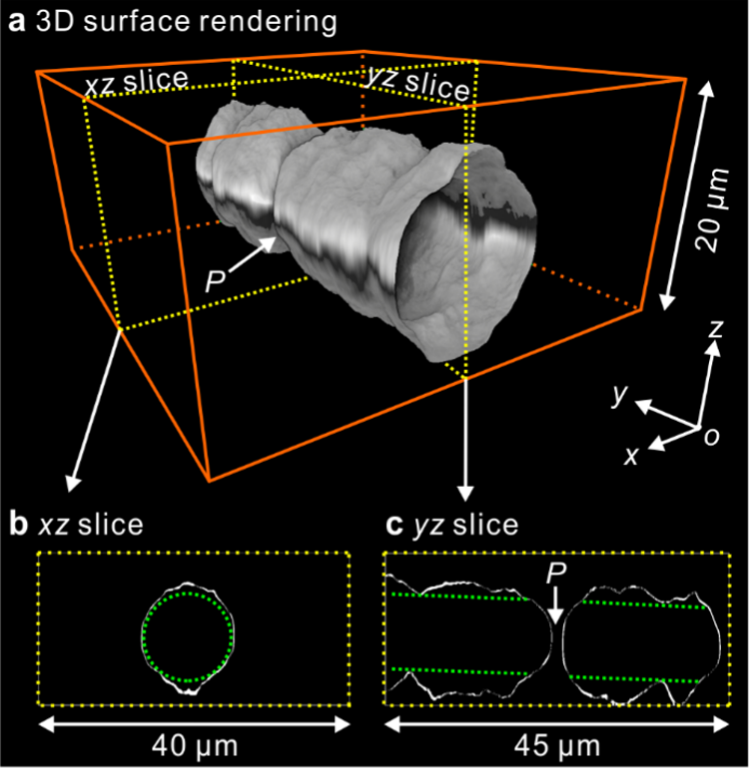
3D images of a PLA cylindrite in a 20 μm film induced by parallel plates shear. The sample was crystallized at 130 °C for ∼6 min and then quenched in ice water. (a) 3D surface rendering. (b) and (c) Vertical *xz*- and *yz*-slices indicated by the yellow dotted lines in (a), respectively.

Prompted by our recent findings of the effects of surface proximity on the growth of spherulite,^45^ we decided to investigate the effect of film thickness on the morphology of the cylindrites. Thus, cylindrites grown in a thinner film (20 μm) were then observed by 2PCM. To obtain their full shape, PLA cylindrites were grown at 130 °C for only ∼6 min to avoid their collision with the top and bottom film surfaces. As shown in Figure 5a, the cylindrite thus obtained has a ridge jutting out both at the top and bottom where the cylindrite is closest to the polymer–substrate interface (see Supplementary Video 1). Again, the cylindrite is tilted about 2 degrees from the top surface plane, suggesting that its axis follows the trajectory of a moving foreign particle under shear flow. Figure 5b,c, showing the vertical *xz*- and *yz*-slices, compares the sections across and along the cylindrite with a reference circle and two parallel lines, respectively. It seems that the closer to the surface, the greater the protrusion of the cylindrite toward it. Similar to the observation of “nonspherical spherulites” observed in our recent 3D study,^45^ this unusual phenomenon is the consequence of the anomalies in the local stress and flow fields induced by negative pressure ahead of the growth front.^63,64^ More unsuspected results of such negative pressure are described further below.

Interestingly, there seems to be a short discontinuity and narrowing of the cylindrite halfway along its length at point *P*. Since shearing was done by hand, there has probably been a short hiatus at the point when the particle reaches point *P* and where the row nucleus was broken.

### 3.3 Fiber Pull Induced Cylindrites

**Figure 6:**
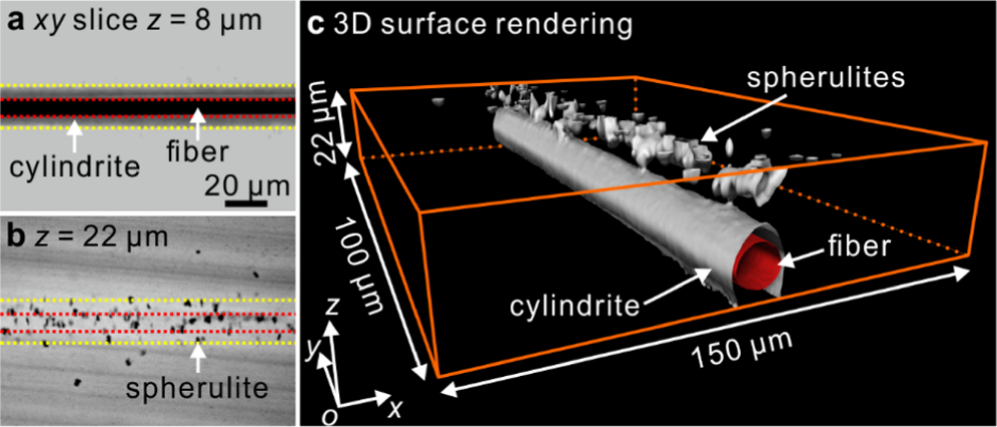
3D images of a PLA cylindrite in 22 μm film induced by fiber pull shear flow. (a, b) *xy*-slices, crystallized at 130 °C for ∼2 min, then quenched in ice water. The contour of the fiber and cylindrite are highlighted by red and yellow dotted lines, respectively. (c) 3D surface rendering, the outer surface of the glass fiber is shown in red to distinguish it from the surface of the PLA cylindrite.

We now turn to PLA cylindrites induced by pulling a glass fiber through the melt. Figure 6a is a *xy*-slice at *z* = 8 μm cutting right through the middle of the fiber. The dark stripe is the fiber and the two surrounding gray stripes on each side is the PLA cylindrite. The diameters measured from the *xy*-slice at *z* = 8 μm are 9.2 ± 0.3 μm for fiber and 4.1 ± 0.5 μm for the full outer diameter of cylindrite. While Figure 5 reminds us that the cylindrite may not be a perfect cylinder, this one is as close to perfection as it gets. Since the fiber is continuous and uniform, unevenness in the pulling rate would cause no unevenness in the cylindrite.

Moving to the top surface of this sample (*xy*-slice at *z* = 22 μm, Figure 6b), we were surprised to find a row of small spherulites, 3.0 ± 0.4 μm in diameter, aligned along the fiber; the red dotted lines are contours of the adjacent fiber. The formation of a large number of small spherulites along the fiber is undoubtedly related, directly or indirectly, to the shear flow induced by fiber pull, as only a few spherulites are seen to have nucleated in the melt outside the yellow dotted lines delineating the contours of the underlying cylindrite at *z* = 8 μm. The question is whether (i) those spherulites are induced directly by the shear flow or (ii) indirectly by the negative pressure at the growth front of the PLA cylindrite. In the former case (i), one would expect the spherulite nucleation density to increase close to the fiber due to the sharp increase in shear rate.^55,62^ On the contrary, the situation actually reminds us of the behavior of spherulite growth in a quiescent melt film of nanoparticle-loaded PLA.^45^ There we have seen how spherulite nucleation is replicated at the opposite surface of the film and postulated that spherulite growth creates negative pressure particularly pronounced at the opposite polymer–glass interface, which initiates copycat nucleation at that interface. Hence, we favor the second explanation (ii) of the currently observed row of spherulites at the surface opposite the pulled fiber. Except in the present case, it is the growing cylindrite rather than spherulites that created the negative pressure at the nearby film surface. Incidentally, most small spherulites and PLA cylindrite are kept some distance apart, as shown in Figure 6c, which further supports the second scenario (ii) of an indirect rather than direct link between fiber pull and the appearance of the row of spherulites (see supporting video 2). We emphasize that for the detection and explanation of these phenomena, 3D imaging is indispensable.

**Figure 7:**
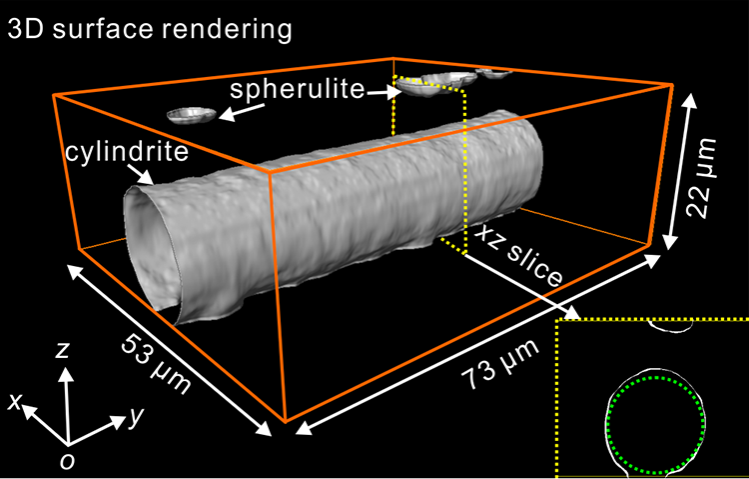
3D image of a PLA cylindrite induced by fiber pull, where the glass fiber was pulled out of the 22 μm film, crystallized at 130 °C for ∼6 min, then quenched in ice water. The inset is a vertical *xz*-slice along yellow dotted lines.

In the next experiment, a PLA cylindrite induced by fiber pull in a 22 μm film is observed. Here, the fiber was pulled out of the matrix completely before cooling to the crystallization temperature. As shown in Figure 7, the cylindrite with a lateral (*xy*-plane) diameter of 13.2 ± 0.4 μm is situated off the film center, so the bottom part of the cylindrite collides with the bottom film surface, while the top part is still about 6.9 μm away from the top surface. The inset *xz*-slice shows that the cylindrite cross section is elongated along the *z*-direction in comparison with a reference circle (green dotted line). Again, a row of spherulites of 4.9 ± 0.5 μm diameter is nucleated at the top film surface, aligned along the cylindrite axis. This observation is consistent with the previous one where the fiber was left inside the matrix (Figure 6). Thus, it indicates that leaving or removing the glass fiber is not a major factor determining the morphology. However, we note that the number of spherulites in the current case where fiber was pulled out of the matrix is much smaller than when the fiber stays in. This can be attributed to the fact that the space between the growing cylindrite and film surfaces in the current case is larger than when the fiber stays in. The larger space in the current case reduces the negative pressure, resulting in a lower density of spherulites.

**Figure 8:**
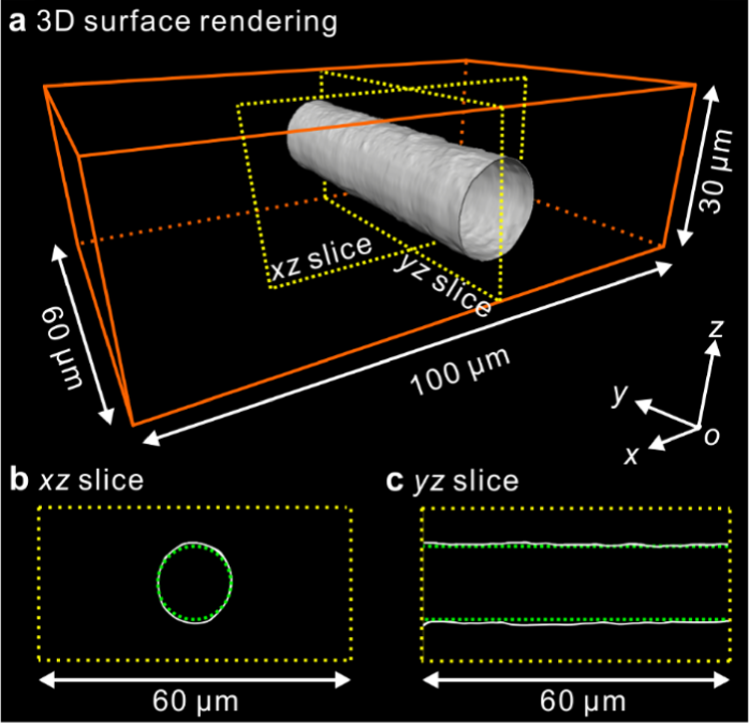
3D images of a PLA cylindrite induced by fiber pull shear, where the glass fiber was pulled out of the 30 μm film, subsequently crystallized at 130 °C for ∼6 min, then quenched in ice water. (a) 3D surface rendering. (b) and (c) Vertical *xz*- and *yz*-slices along yellow dotted lines in (a), respectively.

A further experiment was performed with a 30 μm film. The fiber was also pulled out of the matrix. As shown in Figure 8a, the spherulites are missing, and only the cylindrite is observed. The shape of the outer surface of the cylindrite is again close to that of a perfect cylinder (see Supporting Video 3). However, comparisons with a reference circle in the *xz*-slice (Figure 8b), and with the parallel straight outlines in the *yz*-slice (Figure 8c), reveal that the cylindrite is still slightly extended along *z* as the top and bottom surfaces of the cylindrite approach the film surface. The deformation of the cylindrite is much smaller than that of the cylindrite grown in the 22 μm film with or without the fiber still inside (Figures 6 and 7). A larger film thickness of 30 μm is probably the main reason for the weaker cylindrite deformation.

### 3.4 Effect of Shear History and Environment on the 3D Shape of Cylindrites

In the case where a cylindrite had formed through the action of a traveling particle, its shape can be highly irregular, reflecting the progress of the particle and the general shear history of the system (see Figure 5). At higher shear rates, where laminar flow is replaced by turbulent flow, such a postmortem study of 3D cylindrite shapes may provide information on the flow of melt and of the nucleating particles it had carried. The exact shape of the cylindrite will reveal details not only of the particle trajectory but also of its speed and speed fluctuations; see, e.g., point *P* in Figure 5a,c which indicates the stagnation of flow. One can contemplate extending such experiments to systems with added particles of controlled shape and size that are designed to induce row nucleation. Similarly, varying the shear rate in experiments with more controlled rheological conditions would be a natural extension of such studies.

**Figure 9:**
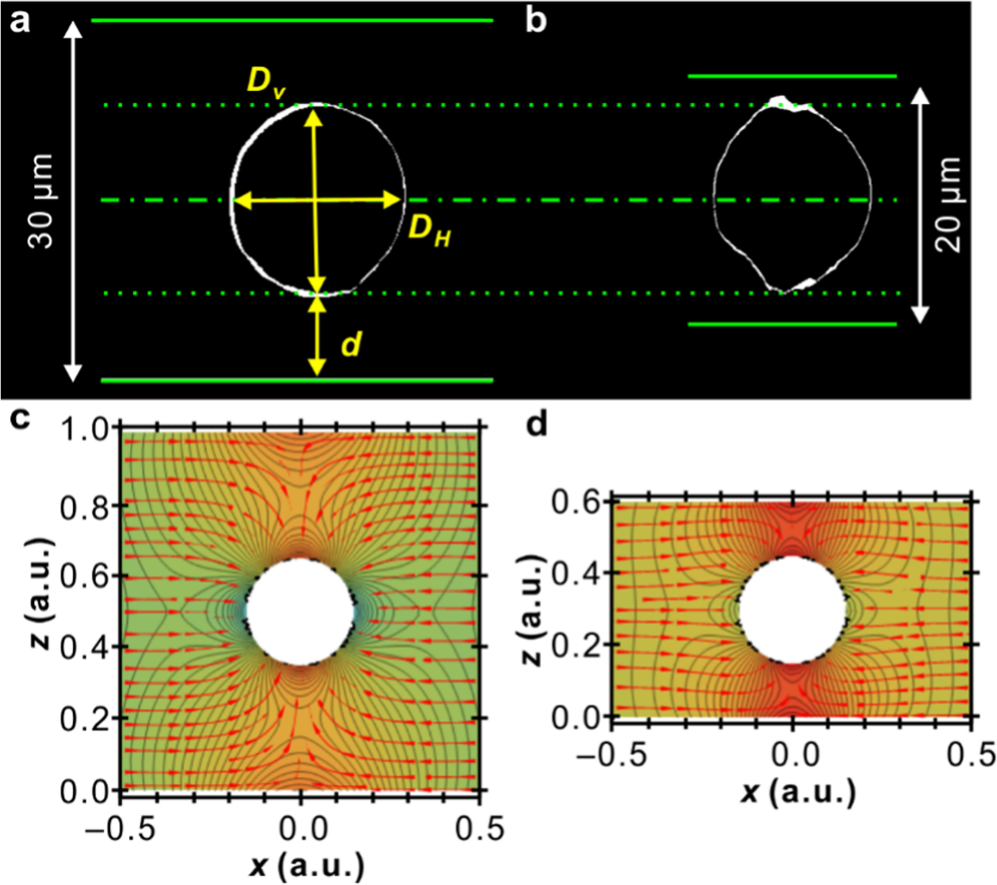
(a, b) Vertical slices of PLA cylindrites with a similar size grown in 30 and 20 μm thick films, respectively. The corresponding diameters along the *z*-plane (*D*_V_) and in the *xy*-plane (*D*_H_), and the shortest distance to the polymer–glass interface (*d*) are defined. (c, d) 2D finite element simulation of flow and pressure around a growing cylindrite. The cylindrite is represented by a circular boundary with a constant inflow rate, halfway between the top and the bottom polymer–glass interfaces. Black lines are the isobars, red arrows are the flow lines, and the background color, from turquoise via yellow to red, shows increasing negative pressure.

Where the flow is laminar and more uniform, particularly in the case of pulling long uniform fibers through the melt, as expected, the cylindrites are more regular in shape, as seen in Figures 6–8. Since they grow laterally from the row-nucleated fibrillar core through epitaxial lamellar crystallization, they would be reasonably expected to have smooth envelopes of cylindrical symmetry. Figure 9a,b shows the cross sections of PLA cylindrites grown in 30 and 20 μm thick films, respectively. Although the two cylindrites have similar sizes, their shapes are different. The aspect ratios of their cross sections (*D*_V_/*D*_H_) are about 1.08 and 1.21, respectively. Such an elliptical distortion of cylindrites is rooted in the same dynamics that caused the formation of spherulites of prolate spheroid shape reported in ref (45). In that work, the distortion was attributed to highly negative pressure building up at the growth front as well as at the polymer–substrate interface nearest to the spherulite. The negative pressure accelerated the growth of the spherulite toward the surface and in some cases also triggered the nucleation of a new spherulite at the surface. These phenomena were found to be particularly pronounced in PLA-containing silica nanoparticles, which acted to additionally restrict chain mobility and thus hinder the refilling of cavities caused by volume contraction. As in the case of spherulites,^45^ here we find that a smaller film thickness leads to greater distortion of the cylindrites. In fact, cylindrites are more prone to such a distortion than spherulites, since a negative pressure cavity is spread along a line, while with spherulites it is localized, centered around a spot; while the access for the replenishing polymer in the spherulite case will be 3D, in the case of the line cavity along a cylindrite, it will be only 2D. This explains why we see growth acceleration and nucleation of an extra row of spherulites at the surface opposite (Figures 6 and 7) in the case of cylindrites when using neat polymer, whereas the equivalent pronounced effects in spherulites were seen only in a nanocomposite. We note that the same PLA polymer was used in both studies.

Figures 9c and 8d show a 2D finite element simulation of negative pressure and fluid flow around a growing cylindrite in a box with different heights. In agreement with the above discussion, the negative pressure is considerably more severe in the thinner box, where the solid surface is closer to the growing cylindrite. The simulated stress distribution also explains why a row of spherulites nucleates at the proximal polymer–substrate interface in the 22 μm thick film, as observed in Figures 6 and 7.

## 4 Conclusions

Using fluorescence microscopy and two-photon confocal laser scanning microscopy, we have succeeded in obtaining 3D images of shear-induced cylindrites in PLA. Generally, the images confirm the expected cylindrical 3D shape of the cylindrites but also reveal conspicuous deviations as well as unexpected features of the surrounding morphology. The three significant findings are: (1) the circular cross section of a cylindrite is deformed into an ellipse extended toward the film–substrate interface if the distance between the cylindrite surface and the film surface is less than a critical value; (2) a row of spherulites was found to nucleate at the polymer–substrate interface closest to the growing cylindrite, aligned along the fiber axis when the melt is sheared by fiber pull; (3) while the recently observed analogous phenomena for spherulite growth were found only in PLA loaded with silica nanoparticles, here in the case of cylindrites they are clearly seen even in the neat polymer. Additionally, in the case of shear by sliding plates and the cylindrites being initiated by moving foreign particles, the 3D shape of the cylindrite reflects the discontinuities and unevenness of the shearing process. The observed ellipticity of the cylindrite cross section is due to the stress-induced acceleration of growth near the surface, attributed to the negative pressure buildup at the growth front and at the polymer–substrate interface opposite. The work has demonstrated that 3D morphology is not always as simple as implied by 2D micrographs. There is considerable scope for extended use of the technique in studies of morphology induced by a wide variety of flow and thermal conditions. Thus, 3D imaging of semicrystalline morphology is a powerful additional tool to be used in both polymer science and industry.

## Supporting Information Available

Shear rate of fiber pull experiments; a brief description of 2PCM setup; calculation of spatial resolution of 2PCM; POM observations of PLA crystallization in the vicinity of untouched glass fiber (PDF)
Elliptical cylindrite in a 20 μm film induced by parallel plates shear (MP4)
Row of spherulites aligned along the elliptical cylindrite in 22 μm film induced by fiber pull (MP4)
Cylindrite in 30 μm film after the fiber was pulled out completely (MP4)
