# Supplementary material for: Morphology of Shear-Induced Polymer Cylindrites Revealed by 3D Optical Imaging
Source: Macromolecules. 2022 Nov 10;56(1):198–206. doi: 10.1021/acs.macromol.2c01433 (PMC9835984; doi:10.1021/acs.macromol.2c01433)
Supplement: Supplementary file 1 — ma2c01433_si_001.pdf [file ma2c01433_si_001.pdf]

Supporting Information for:

# Morphology of shear-induced polymer cylindrites revealed by 3D optical imaging

*Shu-Gui Yang,[†,*] Liang-Qing Zhang,[‡] Jiaming Cui,[†] Xiang-bing Zeng,[§] Baolin Guo,[#] Feng Liu,[†] Goran Ungar[†,§,*]*

[†] Shaanxi International Research Center for Soft Materials, State Key Laboratory for Mechanical Behaviour of Materials, Xi'an Jiaotong University, Xi'an 710049, China

[‡] College of Material Science and Engineering, Xi'an University of Science and Technology, Xi'an 710054, China

[§] Department of Materials Science and Engineering, University of Sheffield, Sheffield S1 3JD, UK

[#] State Key Laboratory for Mechanical Behavior of Materials, Frontier Institute of Science and Technology, Xi'an Jiaotong University, Xi'an 710049, China

### S1. Shear rate of fiber pull experiments

The calculated shear rate profile of the fiber pull experiments is shown in **Figure S1**. The shear rate in both experiments was above 500 $s^{-1}$ at the interface, while it dramatically decreased moving away from the fiber.

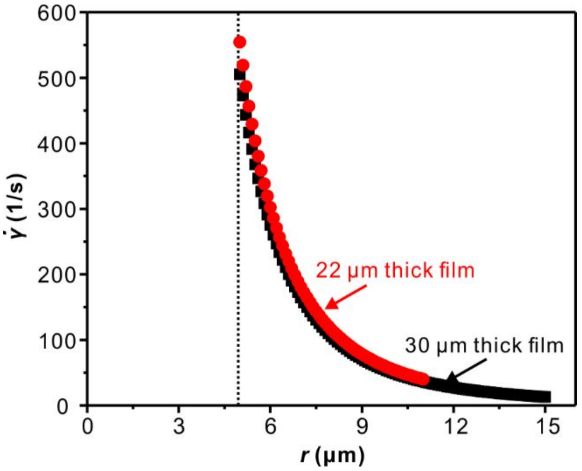


**Figure S1.** Calculated shear rates plotted as a function of the distance from the fiber axis.

### S2. Description of two-photon confocal laser-scanning microscopy (2PCM) setup

As shown in **Figure S2**, the laser beam (Chameleon femto-second pulsed laser) with a wavelength of 1000 nm was reflected by a dichroic mirror (HFT KP 650), and focused by an oil-immersion objective (EC Plan-Neofluar 40×/1.30 Oil DIC) to a small confocal volume in the sample. The laser was then absorbed by NR (excitation). After absorption, the fluorescence from NR (i.e., the fluorescence < 650 nm) was collected by the objective and passed through the dichroic mirror. The fluorescence passed through a beam splitter (NFT 545) and a band pass filter (BP 565-615) in sequence, and was captured by the camera.

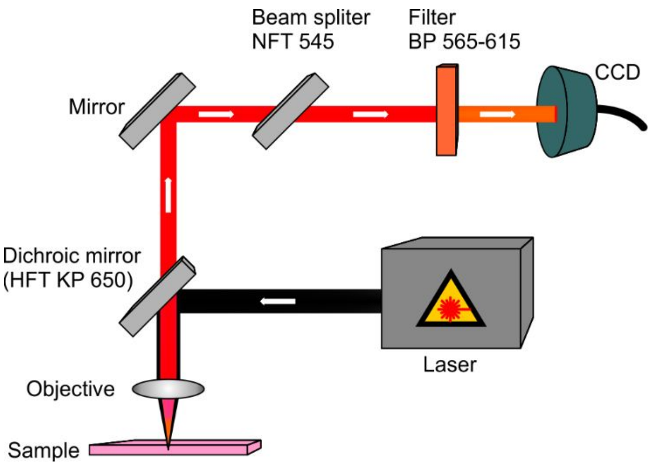


**Figure S2.** Schematic of 2PCM setup.

## S3. Resolution of 2PCM

The resolution in the lateral ($\Delta xy$) and axial ($\Delta z$) directions provided by 2PCM are dependent on the numerical aperture ($NA$) of the microscope objective, the refractive index of the sample ($n$), and the wavelength of excitation laser ($\lambda$).[S1-S3]

$$\Delta xy \approx \frac{1.22\lambda}{2\sqrt{2}NA} \tag{S1}$$

$$\Delta z \approx \frac{2n\lambda}{\sqrt{2}NA^2} \tag{S2}$$

The refractive index of PLA is about 1.4. The calculated $\Delta xy$ and $\Delta z$ are ~332 nm and ~1172 nm, respectively.

## S4. Polarized optical microscopy (POM) observations of PLA crystallization around untouched glass fiber

**Figure S3** shows POM images of Nile Red-doped PLA isothermally crystallized at 130 °C. The imbedded glass fiber was untouched, and there is no sign of increase in nucleation density around the fiber. The results demonstrate that the glass fiber has no nucleating ability for PLA

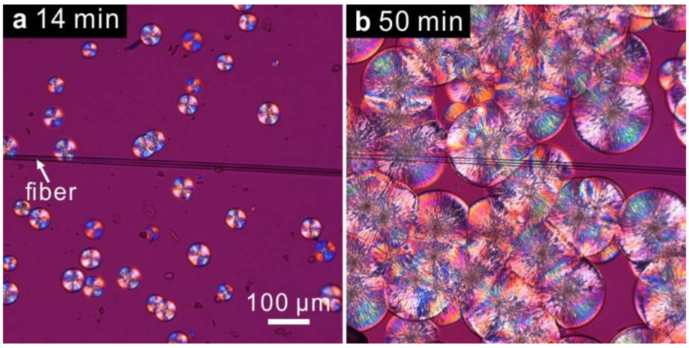


**Figure S3.** POM with a full-wave (λ) plate of NR-doped PLA isothermally crystallized at 130 °C for (a) 14 min and (b) 50 min. The imbedded glass fiber is untouched.

References

[S1] Muller, M., *Introduction to confocal fluorescence microscopy*. SPIE press: **2006**; 2nd ed.

[S2] Denk, W.; Strickler, J. H.; Webb, W. W., Two-Photon Laser Scanning Fluorescence Microscopy. *Science* **1990**, *248* (4951), 73-76.

[S3] Huang, B.; Bates, M.; Zhuang, X., Super-Resolution Fluorescence Microscopy. *Annu. Rev. Biochem.* **2009**, *78* (1), 993-1016.
